# In response: “A novel cause of rebreathing carbon dioxide related to removed CLIC-seal on the Dräger Apollo© Anesthesia Machine” from B. Nikman et al. in this issue of JCMC

**DOI:** 10.1007/s10877-018-0162-z

**Published:** 2018-05-31

**Authors:** Hans Ulrich Schüler, Björn Goldbeck, David Karchner

**Affiliations:** 1 Drägerwerk AG & Co KGaA, Lübeck, Germany; 2 Draeger Medical, Inc., Telford, PA USA

We would like to thank the Editor of this Journal for giving us as the manufacturer the opportunity to respond to this presented case of a missing rubber seal ring leading to increased levels of etCO_2_.

The authors describe two cases of laparoscopy cholecystectomy (1). They describe, that the first case started uneventful. Later on during the case, they observed increased FiCO_2_ values; therefore during the case they removed the CLIC absorber from it’s fitting system (CLIC adapter), and shook it. After re-installing the same CLIC absorber, they observed, that the FiCO_2_ values remained high even after shaking the absorber. Therefore, they decided to remove the absorber completely, and installed a new one. However, after installing the new CLIC absorber, they observed increased FiCO_2_ levels of higher than 10.

The CLIC adapter system is an optional feature for all Dräger anesthesia workstations to allow for fast and easy exchange of the entire pre-filled soda lime canister, without the need to empty and refill a traditional soda lime canister.

**Fig. 1:**
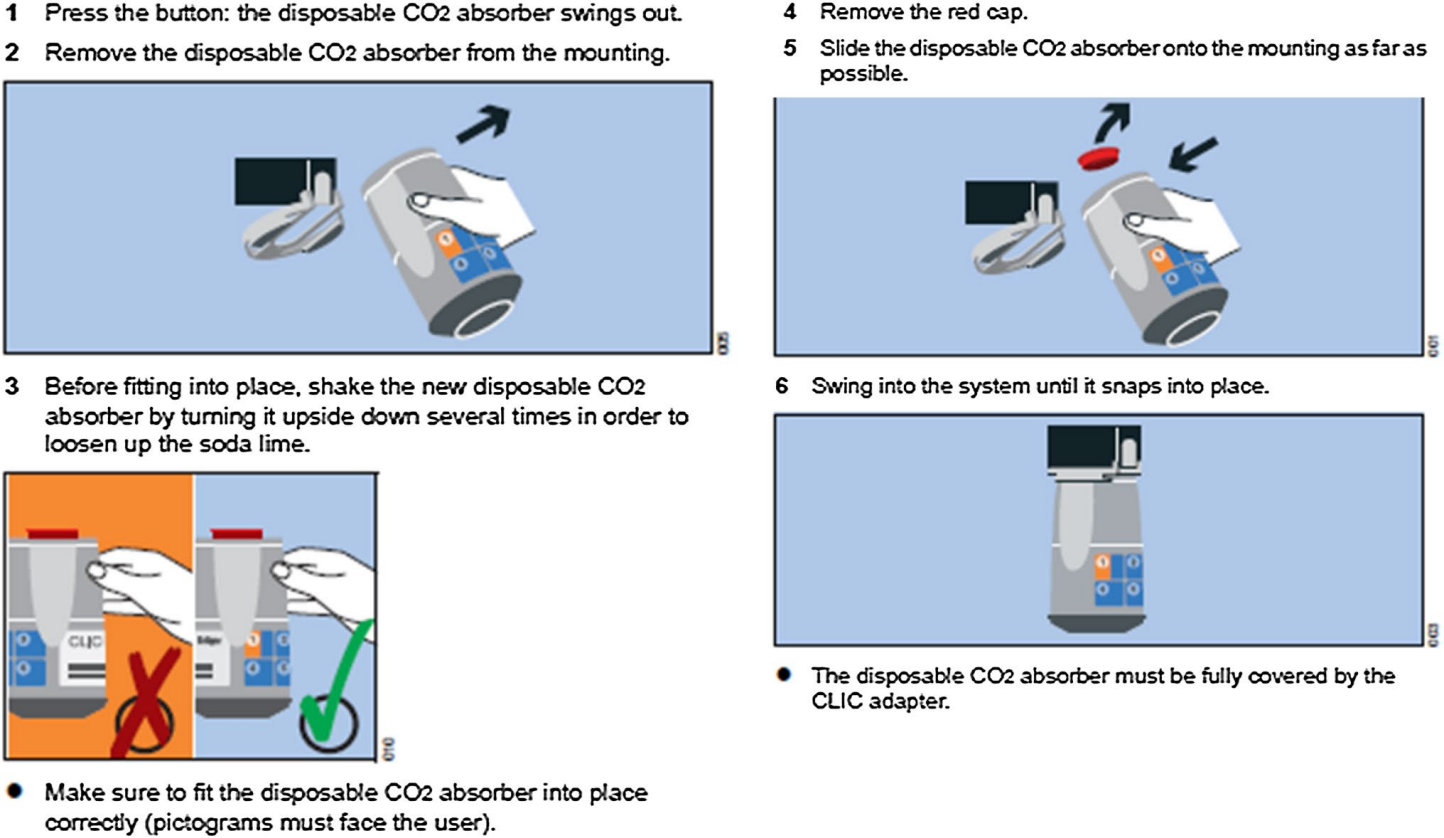
Extract from the Instructions for Use (CLIC absorber 800+/CLIC adapter): steps to exchange the CLIC absorber

In order to exchange this CLIC absorber, the following steps have to be taken, which are also described in the relevant Instructions for Use of the CLIC absorber 800+/CLIC adapter, and also put as an explanatory label on each CLIC absorber itself (Fig. 1).

**Fig. 2:**
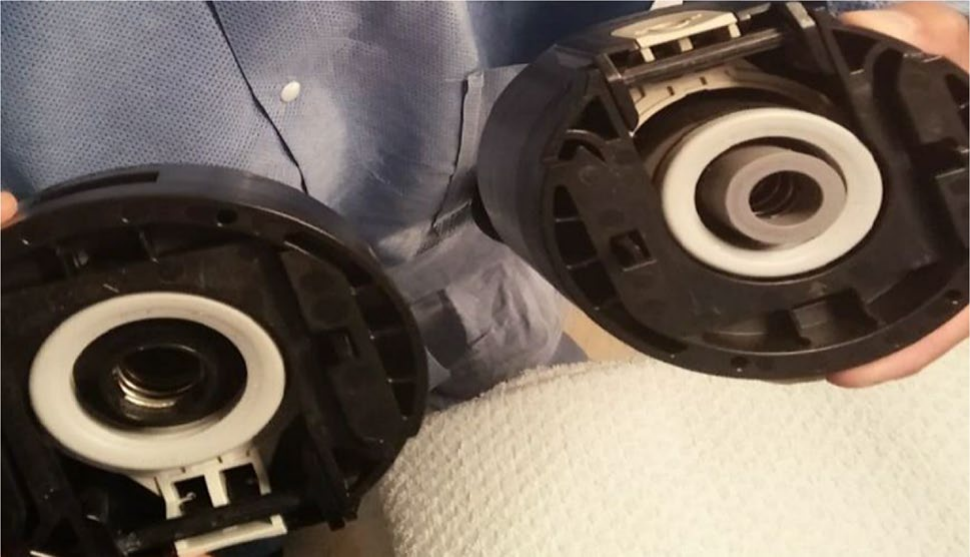
Missing seal ring on the left side CLIC adapter

The authors describe, that upon observation they found a seal ring from the CLIC adapter missing (Fig. 2).

**Fig. 3:**
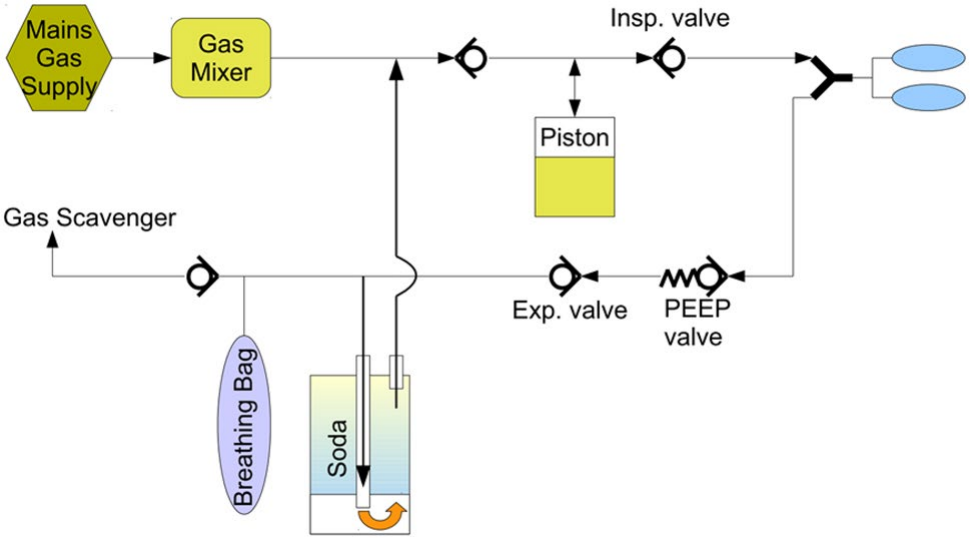
Gas flow inside the Apollo breathing system, with proper positioned seal ring

This seal ring is an integrated part of the CLIC adapter, and not intended to be removed, neither during cleaning nor during any other procedure. The CLIC adapter is maintenance free and needs to be exchanged after 4 years (see Instructions of Use CLIC absorber 800+/CLIC adapter). The seal ring in the middle of the CLIC adapter is designed to be positioned firmly inside the CLIC adapter. It is required in order to guide the gas flow inside the CLIC absorber in the specified way (see Fig. 3).

**Fig. 4:**
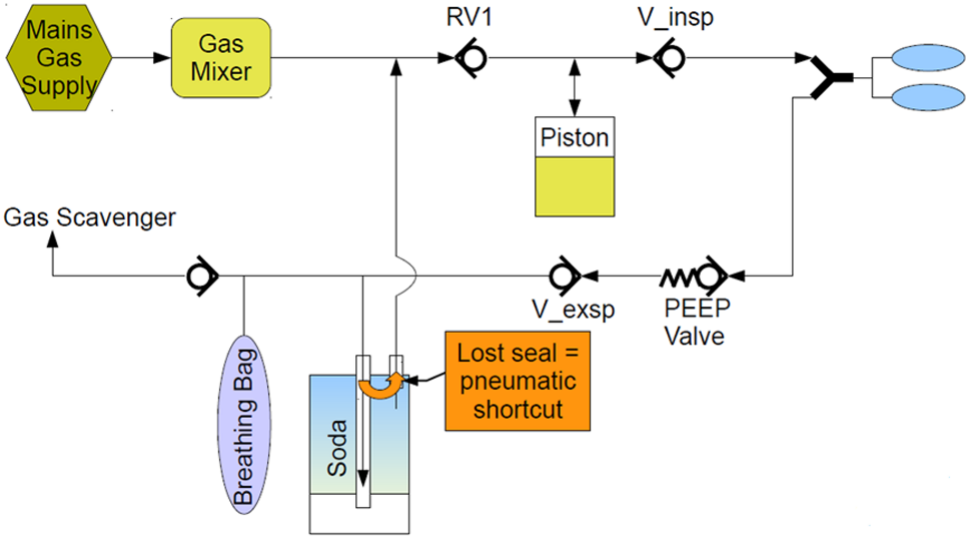
Schematic of the breathing system with missing seal ring: pneumatic short cut inside the CLIC absorber which leads to no CO_2_ removal

The seal ring is specified, tested, and validated to work in the specified way, to guide the gas flow through the filled CLIC absorber in order to bind the exhaled CO_2_ from the patient. In case the seal ring is missing, there will be an internal leakage, creating a “pneumatic short cut” inside the CLIC absorber. In this case no CO_2_ will be removed, as the CO_2_ enriched gas flows from the patient and breathing bag do not pass the soda lime inside the absorber (see Fig. 4).

Therefore we can assume the following: The reported symptom was most likely caused by a missing rubber seal, which is part of the CLIC adapter system and located on the bottom side of the CLIC adapter, facing to the CLIC absorber. The function of this seal ring is to guide the gas flow through the absorber such, that the gas passes the soda lime, and to avoid a direct connection between inlet and outlet port of the absorber canister. It is an integral part of the CLIC system and must not be removed by the user.

Over the life of the Apollo, we have received worldwide only a small number of reports where this seal ring was lost/displaced. The cause was typically due to high forces and wrong usage of the CLIC absorber and CLIC mechanism when closing the CLIC, or due to the health institution incorrectly assembling the CLIC adapter following preventive maintenance/cleaning, often in combination when the CLIC absorber did not get exchanged after 4 years, and plastic material of the seal ring got aged.

Prerequisite for a successful device check is that the workstation has been prepared and assembled correctly. The user has to acknowledge the completeness of the breathing system as part of the checklist implemented in the device software prior to start the automatic device check. The CLIC adapter with seal ring is part of the breathing system. This procedure shall assure that the breathing system is complete prior to operation.

If the inner seal ring of the CLIC adapter is missing, as in the described case, the integrated gas measurement of the device ensures that the high inspiratory CO_2_ concentrations are detected and alarmed immediately. One cause for this event may be that there is an internal leak or fault in the breathing system, as stated in the Instructions for Use of the Dräger Apollo© Workstation, in the chapter “Fault-Cause-Remedy”. A remedy to this cause is to replace the breathing system which includes the CLIC adapter.

To assist our users and further reduce the likelihood of such use failures, Dräger made the following changes to significantly reduce the opportunity of this finding reoccurring:

- In the middle of 2008, Dräger migrated from the “old style” CLIC adapter to the “new style” CLIC adapter. The significance of this migration is that the “new style” CLIC adapter is to be replaced every 4 years, and no longer requires preventive maintenance. This eliminates the concern of the CLIC adapter being reassembled incorrectly by the institution following preventive maintenance.
- At the end of 2013, Dräger further improved the design of CLIC adapter to include adhesive to this seal. This new design eliminates the opportunity for the scenario described by the author to occur via high forces or during cleaning. What this means is:
  - All Apollos shipped with the CLIC option after end of 2013 include this new design which includes the new adhesive to this seal.
  - Apollos shipped with the CLIC option between 2008 and 2013 with the “new style” CLIC adapter should have already migrated to the new design (as long as the 4 year replacement strategy defined by Dräger is being followed).
  - For customers with Apollos including the CLIC option shipped prior to June of 2008 (and have yet to migrate to the “new style” CLIC adapter), a migration could be considered. Again, we have received very few reports similar to this submission, but this migration option is available. The part number for the “new style” CLIC adapter is MX50090.

While Dräger did not have the opportunity to inspect this machine, based on the images provided, the case described by Nikman et al. shows a CLIC adapter manufactured between mid 2008 and end of 2013.

As a summary we very much thank the authors Nikman et al. to bring this case to the attention of the readers of this journal. The submission provided an overview how the device’s integrated gas measurement ensured that the high inspiratory CO_2_ concentrations were detected and alarmed immediately. In addition, the submission highlighted the importance of proper training and servicing according to the Instructions for Use.

